# Synthesis of MnFe_2_O_4_ Spinel on Rusted Q235 Steel Surface and Its Corrosion Resistance Properties

**DOI:** 10.3390/ma17164163

**Published:** 2024-08-22

**Authors:** Bai-Ao Feng, Xu Miao, Ting-An Zhang

**Affiliations:** Key Laboratory of Ecological Metallurgy of Multi-Metal Intergrown Ores of Ministry of Education, Special Metallurgy and Process Engineering Institute, School of Metallurgy, Northeastern University, Shenyang 110819, China; fba19960520@163.com (B.-A.F.); miaoxu0916@163.com (X.M.)

**Keywords:** Q235 steel, composite material, corrosion, spinel, passive films, SEM-EDS, electrochemical impedance spectroscopy

## Abstract

Corrosion of steel is an issue that cannot be ignored in contemporary society. Due to large-scale corrosion, it is urgent to develop a surface treatment process that enhances the corrosion resistance of steel, allowing for application in various scenarios as needed. This study aims to investigate a novel surface treatment process to extend the service life of corroded Q235 steel, reduce its sensitivity to corrosion, and enable its use in multiple environments. This study employs the sol-gel method, using manganese nitrate solutions of varying concentrations to treat the surface of Q235 steel after different electrolysis times. The optimal conditions for precursor preparation were found to be a Mn^2+^ concentration of 0.1 mol/L and an electrolysis time of 2 h. Electrochemical tests using NaCl solutions of different concentrations revealed a significant reduction in the corrosion current for the composite coating based on Q235 steel treated with this method in NaCl solutions with wt.% = 1, 2, 3, 4, 5. Furthermore, the resistance to corrosion was strongest in the NaCl solution with a concentration of 1 wt.% where the corrosion current decreased from 24.8 µA/cm^2^ to 6.79 µA/cm^2^. Additionally, the coating was found to be diffusion-controlled in the early stages of the corrosion process and charge transfer-controlled in the later stages. The MnFe_2_O_4_ spinel coating demonstrated the greatest enhancement in corrosion resistance in the wt.% = 1 NaCl solution.

## 1. Introduction

In recent years, with the development of social productivity, the consumption of metal resources by humans has increased dramatically. A large number of metal products are utilized in human production and daily life, consequently leading to the issue of metal corrosion, which demands significant attention. According to an independent assessment report by the National Association of Corrosion Engineers (NACE), the direct economic loss caused by corrosion worldwide amounts to as much as $250 billion annually, which is equivalent to 3.4% of the global Gross Domestic Product (GDP) [1]. Additionally, corrosion leads to numerous indirect economic losses, including impacts on production safety and the environment [2,3,4,5]. It is estimated that more than 10 million tons of metal rusts in various industries worldwide each year [6].

Carbon steel, due to its excellent processing performance, good thermal conductivity, and outstanding plasticity and toughness, has comprehensive properties and is widely used in various fields. It finds extensive applications in aerospace, shipbuilding, construction steel, and petroleum and natural gas pipelines [7]. However, during usage, corrosion is inevitable [8,9].

To mitigate the corrosion of steel, numerous methods have been studied. Research has shown that carboxymethyl cellulose (CMC) can act as a corrosion inhibitor for Q235 steel in H_2_SO_4_ environments, with its inhibition efficiency increasing with the CMC concentration but decreasing with rising temperatures [10]. This is because CMC exists on the Q235 surface through physical adsorption, and its physical properties change with temperature, affecting its corrosion resistance. Similarly, an imidazoline-based inhibitor has been found to provide high protection efficiency for Q235 steel in NaCl solutions saturated with CO_2_ [11], but its cost and environmental constraints limit its suitability for large-scale applications to Q235 steel. To reduce costs and the environmental impact on corrosion resistance, combining non-metal oxides with Q235 steel has been proposed. Scientists have used the sol-gel method to prepare SiO_2_/ZrO_2_-SiO_2_ composites on the surface of Q235 steel [12], which exhibit excellent corrosion resistance and stability at room temperature. However, these composites tend to undergo brittle fracture at temperatures ≥ 1200 °C. Consequently, metal materials have become a mainstream research direction. It has been found that alloying steel with aluminum results in weathering steel with enhanced corrosion resistance [13]. This steel initially shows good protective performance, but the instability of the passivation film formed on its surface fails to provide long-term protection [14]. Hot-dip coating, an in situ synthesis method, shows promising potential. Studies have found that hot-dip aluminum coating on the inner surface of API X56 and API X70 steel samples forms an adhesive protective layer of aluminum–silicon alloy. This coating surface, through the formation of an Al_2_O_3_ passivation layer, provides strong corrosion resistance to Q235 steel, effectively protecting pipeline steel from marine or seawater corrosion [15].

A novel Al-Mg-Y alloy coating can be synthesized on the surface of Q235 steel through hot-dip coating. In corrosive solutions, the Al-Mg-Y alloy surface can form yttrium-rich structures and new alloy phases containing yttrium, which enhance the alloy’s corrosion resistance [16,17]. However, an excess of yttrium can accelerate the corrosion reaction. Most metal oxides or metal–nonmetal composites exhibit stable properties and strong corrosion resistance. Scientists have developed a new type of Al_2_O_3_-anchored carbon quantum dot modified TiO_2_ nanorod (Al/C/TNRs) photoelectrode material for efficient photo-cathodic protection of Q235 carbon steel under sunlight [18]. However, its high cost limits its application. Research has employed thermal flame spraying to deposit pure aluminum and aluminum/10% Ti_3_SiC_2_ coatings on carbon steel, resulting in excellent corrosion and wear resistance [19]. Nevertheless, given the widespread use of Q235 steel and the significant portion that has already suffered corrosion, this method has limitations. Therefore, it is crucial to develop a low-cost method to treat already corroded Q235 steel, stabilizing its properties and enhancing its corrosion resistance. Research on the treatment of corroded steel has become particularly important as it allows for a gradual reduction in the consumption of human and material resources, as well as the waste of resources caused by replacing long-used steel components. Moreover, this new study not only benefits resource conservation and recycling in steel-dependent industries such as marine, military, construction, and aerospace but also aids in the restoration and long-term preservation of steel artifacts, souvenirs, and historical items in people’s daily lives.

Due to its poor corrosion resistance, Q235 steel, despite being widely used in many industries such as construction, bridges, ships, vehicles, and machinery manufacturing, has a relatively short lifespan and often requires the replacement of products that fail to meet usage requirements due to corrosion. Its corrosion products are mainly loose and porous Fe_2_O_3_, which allows H_2_O and CO_2_ from the air to penetrate and cause more severe internal corrosion. Therefore, surface treatment of already corroded Q235 steel can enhance its corrosion resistance and extend its service life. Currently, most research focuses on substrate modification and the preparation of multilayer composite materials. Scientists have not extensively explored the research area presented in this paper, leaving significant gaps. Previously, some scientists attempted to prepare self-healing fluid materials on steel surfaces to achieve sustained corrosion resistance [20]. A self-healing coating on steel substrate has been obtained by incorporating carbon nanotube (CNT) into the fluid matrix of epoxy resin (EP) or silicone oil (OIL). To further achieve the active corrosion resistance, 1H, 1H, 2H, 2H^−^ perfluorooctyltriethoxysilane (PTES), which can react with the water inside the coating, is added. The fluid self-healing anti-corrosion layer prepared by this method exhibits sustained anti-corrosion performance, but it has poor wear resistance and is highly expensive, with a high dependency on equipment. Therefore, it is necessary to research a low-cost, low-equipment-dependent method that can continuously treat the rust layer on the surface of Q235 steel, thereby enhancing its corrosion resistance.

**Figure 1:**
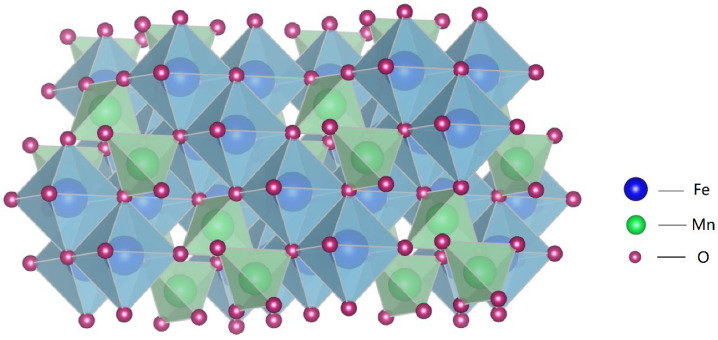
Structure diagram of MnFe_2_O_4_.

The purpose of this study is to explore a method for treating the corrosion layer on the surface of Q235 steel that meets industrial requirements for low cost, reduces dependence on equipment, and has broad applicability. Thus, this study focuses on the corrosion susceptibility of Q235 steel in atmospheric environments, specifically addressing the reality of already corroded Q235 steel. An electrolytic acceleration method is used to simulate the actual application environment of the Q235 steel substrate [21]. To achieve a stable and uniform surface, the sol-gel method is employed [22,23], utilizing different concentrations of Mn(NO_3_)_2_ [24], citric acid, and ammonia as raw materials. Citric acid’s -COOH functional group acts as a reducing agent, while the oxidizing property of NO^3−^ serves as the oxidizing agent. Upon heating, a redox reaction occurs, releasing a significant amount of heat, which facilitates the reaction between the rust layer of the substrate and the Fe^3+^ and Mn^2+^ in the gel layer, ultimately forming a MnFe_2_O_4_ spinel corrosion-resistant coating [25,26,27] (as shown in Figure 1). In different mass fractions of NaCl systems, the open circuit potential, polarization curves, and electrochemical impedance spectroscopy are used to investigate the corrosion susceptibility, electrode reaction parameters during corrosion, corrosion rate, and corrosion mechanisms of untreated and treated Q235 steel. XRD and FT-IR techniques are employed for compositional analysis of the corrosion products. The precursor prepared by the sol-gel method is subjected to high-temperature sintering to obtain the MnFe_2_O_4_ spinel coating, which is then characterized using XRD, FT-IR, and SEM.

This study examines the effects of various process conditions, such as different electrolysis times and Mn^2+^ concentrations, on the MnFe_2_O_4_ spinel coating to determine the optimal process conditions. Different concentrations of NaCl solutions are used to simulate non-uniform application conditions, and the corrosion resistance of the MnFe_2_O_4_ spinel coating is investigated using polarization curves and electrochemical impedance spectroscopy. This research provides crucial scientific evidence for the development of new methods for remediating and enhancing the corrosion resistance of metal surface coatings.

## 2. Methods and Materials

### 2.1. Materials

Q235 steel (10 × 10 × 5 mm) was used as the anode and stainless steel as the cathode. Electrolysis was conducted at room temperature using a DC power (L305SPL, TongMen Electro Tech Co., Dongguan, China) supply with a current density of 1500 A/m^2^ for 60, 90, 120, 150, and 180 min to obtain oxidation layers with varying degrees of reaction. By comparison, the optimal electrolysis time was determined. Prepared Mn(NO_3_)_2_ solutions with concentrations of 0.02 mol/L, 0.04 mol/L, 0.06 mol/L, 0.08 mol/L, and 0.1 mol/L, along with a small amount of citric acid, were ultrasonically mixed evenly. In order to closely approximate the actual industrial production environment and to avoid secondary corrosion caused by the reaction between citric acid and the substrate, ammonia was added to adjust the pH to 6. The electrolyzed Q235 steel was immersed in the prepared solution, followed by water bathing (HH-6, RongHua Instrument Manufacturing Co., Nanjing, China) at 60 °C for 2.0 h and then at 95 °C for 2.0 h. The samples were dried in a vacuum drying oven (DZF-6020, XinMiao Medical Equipment Manufacturing Co., Shanghai, China) at 60 °C for 12 h. The dried samples were then placed in a tube furnace (SK-G08143, Central Electric Furnace Co., Dongguan, China) under an argon atmosphere to maintain vacuum conditions. The calcination was performed at 650 °C with a heating rate of 10 °C/min, and the samples were held at this temperature for 1.0 h, resulting in Q235 steel samples with MnFe_2_O_4_ spinel corrosion-resistant coatings. The reaction is as follows:

(1) $$1.8\mathrm{Mn}({\mathrm{NO}}_{3}{)}_{2}+3.6\mathrm{FeOOH}+C_{6}H_{8}O_{7}\to 1.8{\mathrm{MnFe}}_{2}O_{4}+1.8N_{2}(g)+5.8H_{2}O(g)+6{\mathrm{CO}}_{2}$$

### 2.2. Electrochemical Measurements

#### 2.2.1. Electrode Preparation

A copper wire was soldered to the non-working surface of the Q235 steel using a welding torch and sealed with epoxy resin, leaving an exposed area of 1 cm^2^ for testing. The exposed working surface was sequentially polished using 400, 600, 1000, 1500, 2000, and 3000 grit sandpapers. The surface was then polished to a mirror finish using chromium oxide powder on a metallographic polishing machine (P-2, Huaxing Testing Instruments Co., Jinan, China) and wiped with anhydrous ethanol. The surface morphological patterns of the samples were observed using a scanning electron microscope (SEM, ZEISS Gemini 300, Carl Zeiss AG, Oberkochen, Germany). The samples were characterized by X-ray diffraction (XRD) using a D8 Advance diffractometer (Karlsruhe, Germany) under conditions of 40 kV and 40 mA, 2θ = 10–90°, scan rate of 8°/min, and step length of 0.02°. Each group of samples was tested three times. FT-IR testing was conducted using a Fourier-transform infrared spectrometer (Nicolet IS 50, Thermo Fisher Scientific, Waltham, MA, USA).

#### 2.2.2. Measuring Condition

In this experiment, all electrochemical tests were performed in triplicate using the same set of samples, utilizing an electrochemical workstation (Zennium Pro, Zahner, Kronach, Germany). A three-electrode system was utilized, with Q235 steel serving as the working electrode (dimensions: 10 × 10 × 5 mm), a platinum sheet as the counter electrode (dimensions: 10 × 10 × 1 mm), and a saturated calomel electrode (SCE) filled with saturated KCl solution as the reference electrode. The electrochemical testing conditions were as follows:

Open Circuit Potential (OCP): Testing duration: 7200 s, Frequency: 0.1 Hz. Polarization Curve: Conducted after OCP stabilization for 600 s, potential range: −0.6 V to 1.0 V, scan rate: 0.1 mV/s. Electrochemical Impedance Spectroscopy (EIS): Conducted after OCP stabilization for 600 s, sine wave potential amplitude: 10 mV, frequency range: 100 kHz to 5 mHz, every ten points was sampled five times.

## 3. Results and Discussion

### 3.1. The Impact of Electrolysis Time on the Corrosion Resistance of MnFe_2_O_4_ Spinel Coatings

#### 3.1.1. XRD Analysis of Samples with Different Electrolysis Times

**Figure 2:**
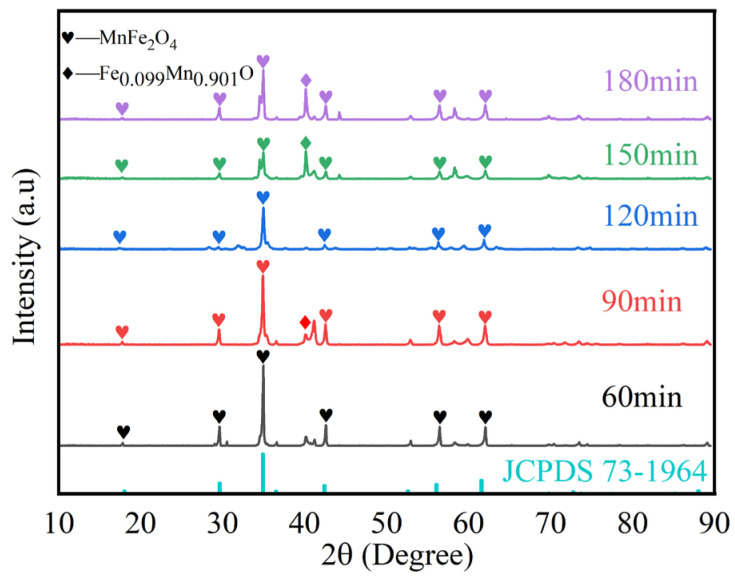
XRD results of samples after 60 min, 90 min, 120 min, 150 min, and 180 min of electrolysis with 0.1 mol/L Mn^2+^ concentrations.

Figure 2 shows the XRD patterns for electrolysis times of 60, 90, 120, 150, and 180 min. Characteristic diffraction peaks corresponding to (111), (220), (311), (400), (511), and (440) are observed, which match the standard MnFe_2_O_4_ pattern (JCPDS 73-1964). As the electrolysis time increases, the intensity of the main peak initially increases and then decreases, indicating that the crystallinity first increases and then decreases. This suggests that the content of spinel-phase MnFe_2_O_4_ increases with electrolysis time initially, reaching a peak at 120 min, and then decreases. The intensity of the impurity peak (FeO)_0.099_(MnO)_0.901_ (JCPDS 77-2362) is higher at electrolysis times of 60, 90, 150, and 180 min compared to 120 min, indicating that the formation of (FeO)_0.099_(MnO)_0.901_ is related to electrolysis time. At electrolysis times of 60, 90, 150, and 180 min, the amount and growth of the non-target product (FeO)_0.099_(MnO)_0.901_ affect the formation of the target product MnFe_2_O_4_. This also explains why the MnFe_2_O_4_ crystals formed at 120 min of electrolysis show the best growth. The XRD results demonstrate that at an electrolysis time of 120 min, the crystallinity of MnFe_2_O_4_ on the surface is the highest, making it the optimal electrolysis time for the formation and accumulation of the spinel.

#### 3.1.2. FT-IR Analysis of Samples with Different Electrolysis Times

**Figure 3:**
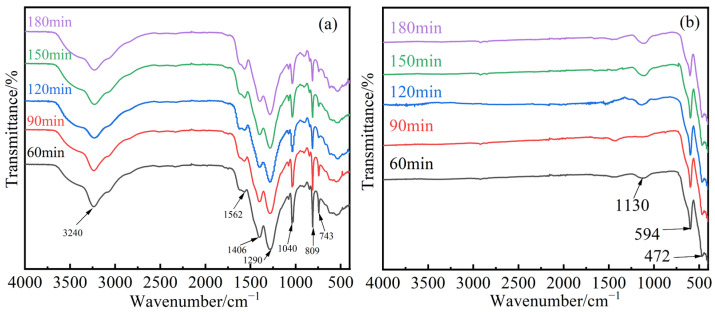
FT-IR results of samples after 60 min, 90 min, 120 min, 150 min, and 180 min of electrolysis with 0.1 mol/L Mn^2+^ concentrations. (**a**) Uncalcined precursor, (**b**) Calcined precursor.

Figure 3a,b shows the FT-IR spectra of the precursors (the precursor is Q235 steel with an oxide layer, electrolyzed for 60, 90, 120, 150, and 180 min under a current of 1500 A/m^2^). This study employs the electrolysis of Q235 steel to simulate the conditions of Q235 steel with a rust layer after prolonged use and calcined samples at different electrolysis times. In Figure 3a, the trends are consistent, indicating that the current density has no effect on the precursor. An absorption peak around 3240 cm^−1^ is observed, corresponding to the stretching vibration of -OH, indicating the presence of a small amount of bound water in the dried samples. The peak near 1562 cm^−1^ is attributed to the stretching vibration of N-O, possibly indicating the presence of NO^3−^ in the precursor. The absorption peak around 1406 cm^−1^ is due to the asymmetric stretching vibration of COO^−^. The peaks near 1290 cm^−1^ and 1040 cm^−1^ correspond to the C-O stretching vibrations in -COOH. The absorption peaks below 1000 cm^−1^ are assigned to the γ_2_ peaks of Fe-OH and Mn-OH.

In Figure 3b, an impurity peak is observed around 1130 cm^−1^ because calcination removes most organic impurities; however, the C-O bond is relatively stable, and calcination does not completely eliminate carbon compounds. Nevertheless, their presence does not affect the subsequent test results, while the characteristic absorption peaks of Mn-O and Fe-O are seen at 594 cm^−1^ and 472 cm^−1^, respectively.

#### 3.1.3. Surface Morphology Analysis of Samples with Different Electrolysis Times

**Figure 4:**
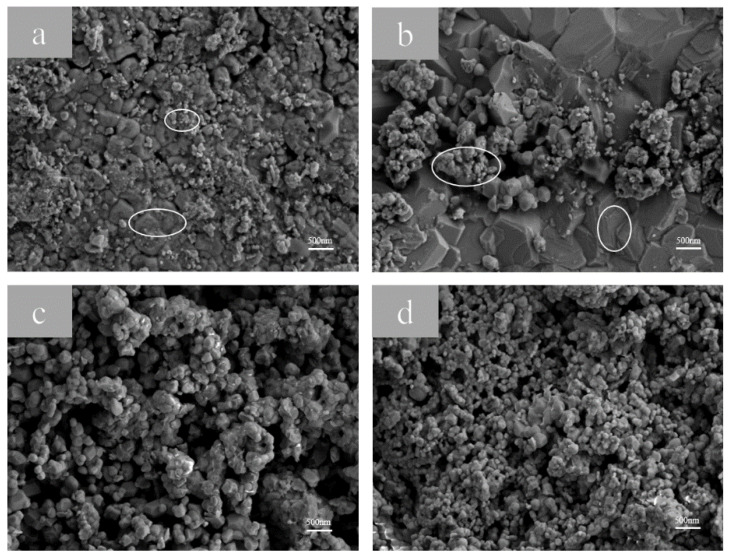
Scanning electron microscope photos of samples after (**a**) 60 min, (**b**) 90 min, (**c**) 150 min, and (**d**) 180 min of electrolysis with 0.1 mol/L Mn^2+^ concentrations. The circles show the characteristic structures of MnFe_2_O_4_ and Fe_3_O_4_.

Figure 4a–d presents the morphological patterns of the samples at electrolysis times of 60, 90, 150, and 180 min, respectively. The morphological patterns of the samples at an electrolysis time of 120 min are shown after. In Figure 4a–d, the number of fine MnFe_2_O_4_ particles on the coating surface initially decreases and then increases [28,29]. In Figure 4a, the few fine MnFe_2_O_4_ particles and the presence of large continuous crystals suggest that the short electrolysis time results in insufficient Fe_3_O_4_ in the rust layer, leading to incomplete reactions. In Figure 4b, a mixed structure of large continuous crystals and MnFe_2_O_4_ particles is observed [30].

**Figure 5:**
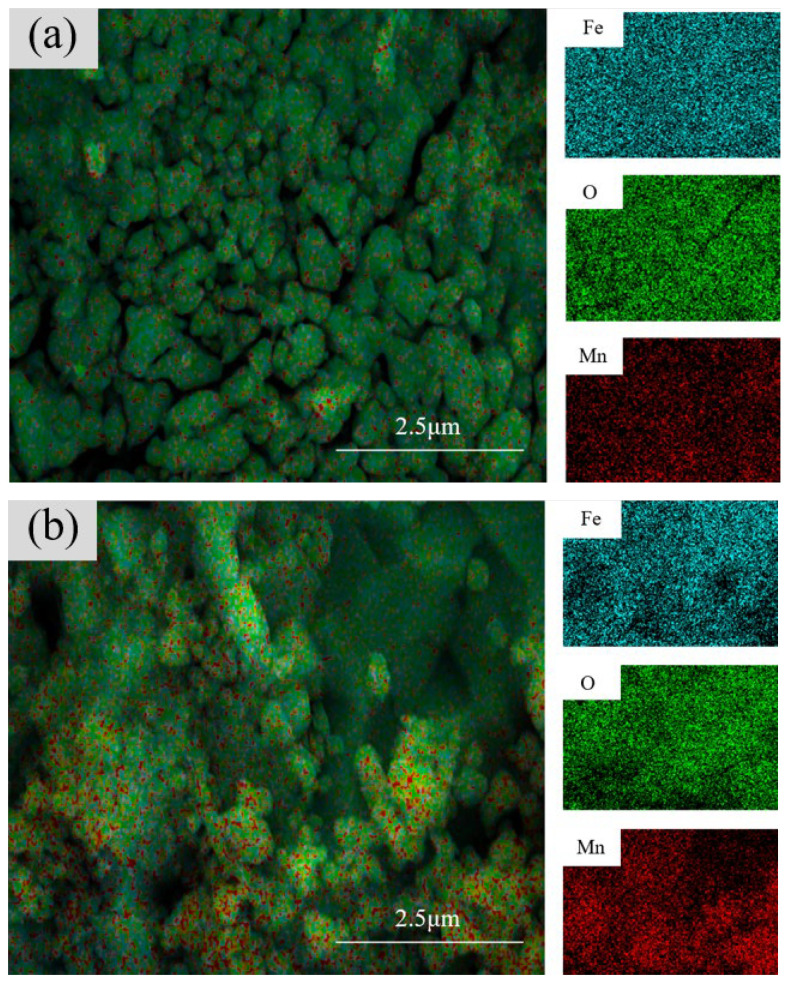

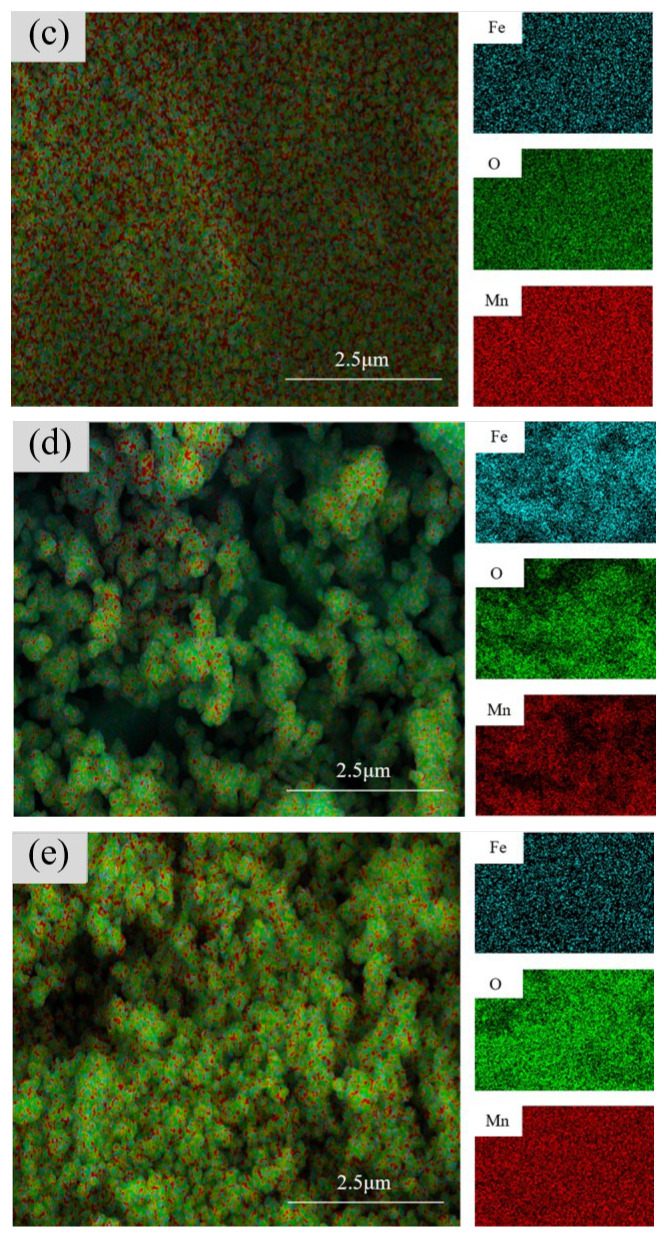
EDS diagram of samples after (**a**) 60 min, (**b**) 90 min, (**c**) 120 min, (**d**) 150 min, and (**e**) 180 min of electrolysis with 0.1 mol/L Mn^2+^ concentrations.

Figure 5a–e presents the EDS maps for electrolysis times of 60, 90, 120, 150, and 180 min, respectively. From Figure 5a, it can be seen that the distribution of Mn atoms is not very uniform, with some areas lacking Mn atoms. The fine spherical aggregates and large crystalline blocks are loosely combined, with a large crack indicating high porosity. In Figure 5b, two different structures are observed: a large crystalline block covered with numerous fine spherical aggregates. This suggests different compositions. The EDS spectrum shows that Mn is barely present in the large crystalline regions, whereas in the spherical aggregate regions, the Mn content matches the overall average, indicating that the different morphologies arise from Mn content variations [31,32]. In Figure 5c, a relatively uniform MnFe_2_O_4_ layer formed on the coating surface. Figure 5d shows many spherical aggregates dispersed separately, resulting in high porosity between them. The morphological patterns of the samples in Figure 4c reveal numerous defects, possibly due to the long electrolysis time causing pits on the Q235 steel surface. Figure 5d demonstrates a uniform distribution of all elements. Combined with the morphological patterns of the sample images, this indicates successful rust layer conversion. The SEM spectra in Figure 5a–e show the appearance and reduction of large crystalline blocks, which are gradually replaced by spherical aggregates. This suggests that during the coating synthesis process, some base materials did not fully convert, and the conversion process likely involves the dissolution and recrystallization of crystals. This process leads to the different morphologies observed, indicating that with increased electrolysis time, the original rust layer more readily converts to MnFe_2_O_4_ in subsequent synthesis steps, resulting in an increased MnFe_2_O_4_ content in the final coating.

#### 3.1.4. Cross-Sectional Morphology Analysis of Samples with Different Electrolysis Times

**Figure 6:**
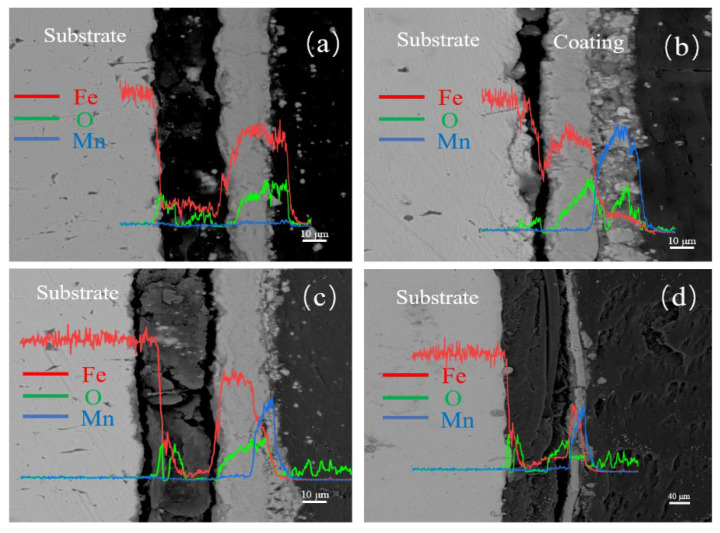
Cross-sectional morphology of samples after (**a**) 60 min, (**b**) 90 min, (**c**) 150 min, and (**d**) 180 min of electrolysis with 0.1 mol/L Mn^2+^ concentrations.

Figure 6 shows the cross-sectional morphologies at different electrolysis times, where Figure 6a, Figure 6b, Figure 6c, and Figure 6d represent electrolysis times of 60, 90, 150, and 180 min, respectively.

From Figure 6a, it can be observed that the area is divided into three layers. The leftmost layer is the substrate. According to the line scan results, the middle layer shows a decrease in Fe atoms and an increase in O atoms, possibly due to partial oxidation of the substrate. The prepared spinel coating is not observed, likely because the electrolysis time was too short, resulting in a thin rust layer and thus the MnFe_2_O_4_ being synthesized only on the surface of the substrate. In Figure 6b, a crack separates the middle layer from the substrate, possibly indicating delamination between the rust layer and the substrate during electrolysis. The O content in the middle layer is increased, and combined with the microstructural characterizations, this region is identified as the rust layer formed during electrolysis. Just to the right of the rust layer, small particles are seen filling in to form a new region, where the Mn content is significantly increased. According to the XRD and FT-IR characterizations, this region is identified as the MnFe_2_O_4_ spinel coating. Figure 6c shows that the spinel coating, located to the far right near the resin, has a low thickness and an uneven distribution, possibly due to defects in the rust layer caused by the prolonged electrolysis time. In Figure 6d, the bonding between the rust layer and the spinel coating is poor. The middle layer appears to be infiltrated by resin, which has penetrated into the substrate and rust layer. Combining the observations from Figure 6a–d, it can be concluded that with increasing electrolysis time, the rust layer initially increases and then decreases. This may be due to poor adhesion between the substrate and the rust layer, leading to delamination when the electrolysis time is too long.

### 3.2. The Effect of Mn^2+^ Concentration on the Corrosion Resistance of MnFe_2_O_4_ Spinel Coatings

#### 3.2.1. XRD Analysis of Samples with Different Mn^2+^ Concentrations

**Figure 7:**
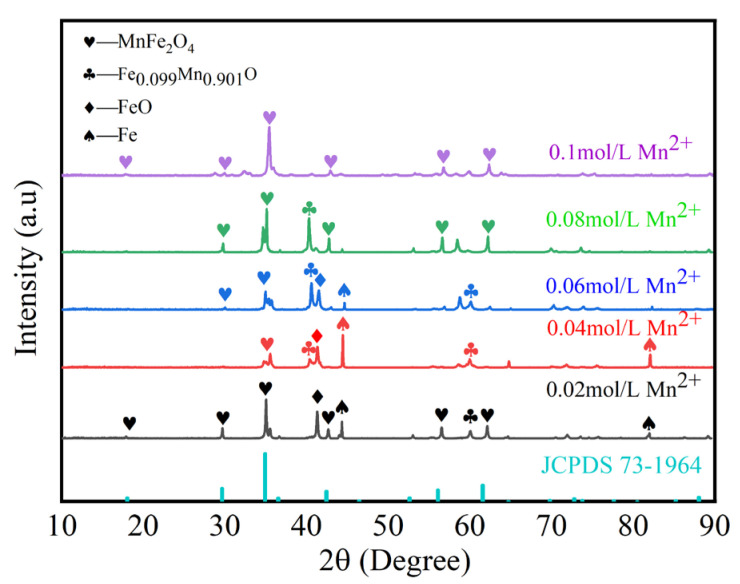
XRD of samples after 120 min of electrolysis with different Mn^2+^ concentrations: 0.02 mol/L, 0.04 mol/L, 0.06 mol/L, 0.08 mol/L, and 0.1 mol/L.

Figure 7 illustrates the effect of different Mn^2+^ concentrations on the preparation of MnFe_2_O_4_ spinel coatings. As shown in the figure, under the condition of 0.02 mol/L Mn^2+^, MnFe_2_O_4_ is not formed, likely due to the Mn^2+^ concentration being too low to initiate the reaction, resulting in Fe_3_O_4_ as the primary phase. As the Mn^2+^ concentration gradually increases, irregular iron-manganese oxides are initially formed, followed by the appearance of characteristic peaks of MnFe_2_O_4_. At 0.1 mol/L Mn^2+^, the characteristic diffraction peaks of (111), (220), (311), (400), (511), and (440) appear, matching the standard MnFe_2_O_4_ pattern (JCPDS: 73-1964). Impurity peaks are almost eliminated, and the diffraction peaks exhibit high intensity and good crystallinity. Therefore, the optimal concentration of Mn^2+^ is determined to be 0.1 mol/L.

#### 3.2.2. FT-IR Analysis of Samples with Different Mn^2+^ Concentrations

**Figure 8:**
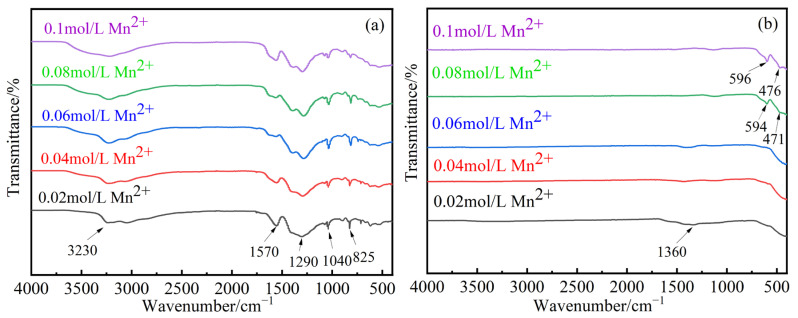
FT-IR of samples after 120 min of electrolysis with different Mn^2+^ concentrations: 0.02 mol/L, 0.04 mol/L, 0.06 mol/L, 0.08 mol/L, and 0.1 mol/L. (**a**) Uncalcined precursor, (**b**) Calcined precursor.

Figure 8a shows the FT-IR spectra of the precursors with different Mn^2+^ concentrations before calcination, and Figure 8b shows the FT-IR spectra after calcination. From Figure 8a, a broad absorption peak near 3230 cm^−1^ is observed, corresponding to the stretching vibration of -OH. The peak around 1570 cm^−1^ corresponds to the stretching vibration of N-O, possibly indicating the presence of NO^3−^ in the precursor. The peaks near 1290 cm^−1^ and 1040 cm^−1^ correspond to the C-O stretching vibrations in -COOH. The absorption peaks below 1000 cm^−1^ are attributed to the γ_2_ peaks of Fe-OH and Mn-OH. As the Mn^2+^ concentration increases, the peak positions remain unchanged, but the widths increase. From Figure 8b, it is evident that MnFe_2_O_4_ exhibits two characteristic peaks in the 400–600 cm^−1^ range, corresponding to the Mn-O and Fe-O absorption peaks. At low concentrations, these characteristic peaks are either absent or have very low transmittance, and there is an impurity peak at 1360 cm^−1^. When the Mn^2+^ concentration is 0.08 mol/L, two characteristic peaks appear around 594 cm^−1^ and 471 cm^−1^, with very few impurity peaks. When the Mn^2+^ concentration is 0.1 mol/L, two characteristic peaks appear near 596 cm^−1^ and 476 cm^−1^, with very few impurity peaks, indicating the formation of MnFe_2_O_4_ spinel at both 0.08 mol/L and 0.1 mol/L, which is consistent with the XRD analysis.

#### 3.2.3. Surface Morphology Analysis of Samples with Different Mn^2+^ Concentrations

**Figure 9:**
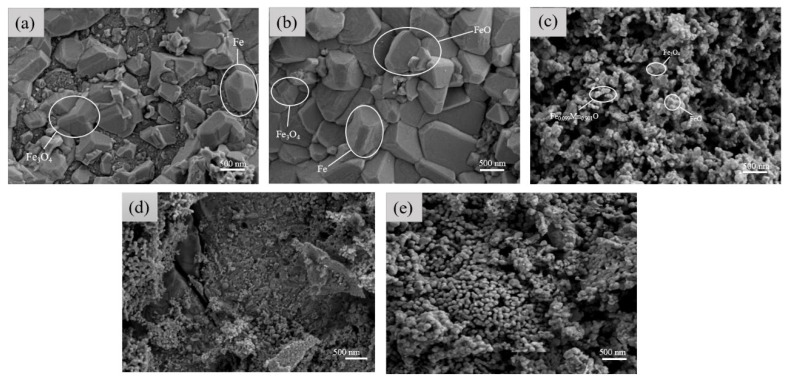
Morphological patterns of samples after 120 min of electrolysis with different Mn^2+^ concentrations: (**a**) 0.02 mol/L, (**b**) 0.04 mol/L, (**c**) 0.06 mol/L, (**d**) 0.08 mol/L, (**e**) 0.1 mol/L.

Figure 9a–e shows the morphological patterns of the samples of MnFe_2_O_4_ spinel coatings prepared with Mn^2+^ concentrations of 0.02, 0.04, 0.06, 0.08, and 0.1 mol/L, respectively. In Figure 9a, two main types of shapes can be observed: the majority are irregular protrusions identified as Fe_3_O_4_, while the less common shapes are three-dimensional convex polygons identified as Fe, which are scattered and disordered. Figure 9b shows, in addition to the previously mentioned shapes, a new layer-like structure identified as FeO. This structure is very smooth and closely bonded [33]. In Figure 9c, the synthesis of iron-manganese oxides begins, but due to the insufficient Mn^2+^ concentration, irregular iron-manganese compounds are formed. The morphological patterns of the samples appear spherical, with uneven surface morphology and noticeable agglomeration [34,35]. Figure 9d shows the formation of MnFe_2_O_4_ spinel with a polygonal surface morphology, but Fe_3_O_4_ is still present, indicating that the reaction is incomplete and that the Mn^2+^ concentration is still insufficient [36]. In Figure 9e, with a Mn^2+^ concentration of 0.1 mol/L, the reaction is complete. The sol-gel prepared MnFe_2_O_4_ shows well-developed polyhedral crystals with sizes around 125 ± 15 nm, exhibiting stacking and slight agglomeration. This is consistent with the microstructure of MnFe_2_O_4_ previously observed by scientists [37,38].

#### 3.2.4. Cross-Sectional Morphology Analysis of Samples with Different Mn^2+^ Concentrations

**Figure 10:**
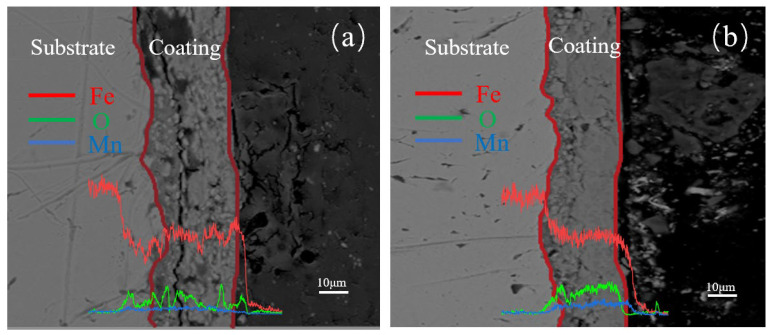
Cross-sectional morphology of samples after 120 min of electrolysis with different Mn^2+^ concentrations: (**a**) 0.08 mol/L, (**b**) 0.1 mol/L.

After a current density of 1500 A/m^2^ for 120 min of electrolysis, Figure 10a,b shows the cross-sectional morphologies of the coatings formed with 0.08 mol/L Mn^2+^ and 0.1 mol/L Mn^2+^, respectively, with line scans included. From Figure 10a, the MnFe_2_O_4_ spinel coating thickness is around 15 μm. The coating exhibits noticeable voids and layering, with the layering located in the middle of the coating, showing distinct differences in morphology above and below this layer. This suggests a separation between Fe_3_O_4_ and MnFe_2_O_4_. The area near the substrate has a finer structure and shows good transition. From Figure 10b, the MnFe_2_O_4_ spinel coating thickness is around 20 μm. The coating contains fewer voids, and near the substrate, there is a visible transition between the substrate and the coating, with interlocking structures. The voids appear as fine particles in the morphological patterns of the samples. This indicates that the coating has a tight bond with the substrate, with a dense and strong exposed surface. Furthermore, the line scan results indicate that the Mn atomic ratio at the 0.08 mol/L Mn^2+^ concentration increases slightly compared to the substrate. However, at the 0.1 mol/L Mn^2+^ concentration, the Mn atomic ratio significantly increases compared to the substrate, indicating the successful preparation of the spinel coating.

### 3.3. Corrosion Resistance Testing of MnFe_2_O_4_ Spinel Coatings

#### 3.3.1. Potentiodynamic Polarization Measurement

**Figure 11:**
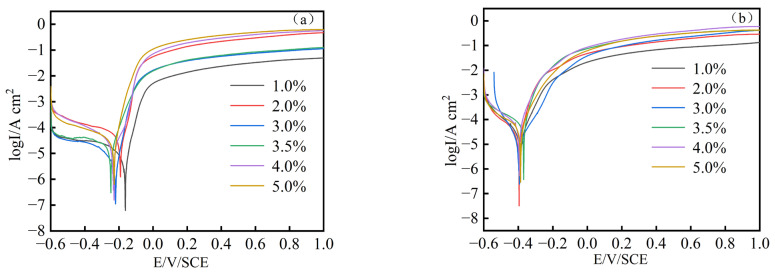
Polarization curve of samples after 120 min of electrolysis with or without coating in NaCl solution with different mass fractions: 1%, 2%, 3%, 3.5%, 4%, and 5%. (**a**) MnFe_2_O_4_ spinel coating, (**b**) Q235 steel.

Figure 11a,b shows the polarization curves of the prepared MnFe_2_O_4_ spinel coating and Q235 steel in NaCl solutions with mass fractions of 1 wt.%, 2 wt.%, 3 wt.%, 3.5 wt.%, 4 wt.%, and 5 wt.%, respectively. The scan rate was 0.1 mV/s. The polarization curves were fitted using Zahner Analysis software (https://zahner.de/products-details/software/zahner-analysis, accessed on 20 August 2024) to calculate the corrosion potential (*E*_corr_), corrosion current density (*I*_corr_), and polarization resistance (*R*_p_) values, as shown in Table 1 and Table 2. From Table 2, it can be seen that the self-corrosion potential of Q235 steel is around −400 mV, while the self-corrosion potential of the MnFe_2_O_4_ spinel coated Q235 steel is around −250 mV, an increase of 150 mV, indicating that the MnFe_2_O_4_ spinel coating reduces the corrosion tendency of Q235 steel. Comparing Table 1 and Table 2, it is evident that the corrosion current of the MnFe_2_O_4_ spinel coated Q235 steel is consistently several times lower than that of Q235 steel in NaCl solutions of different mass fractions. Specifically, the corrosion current density of the MnFe_2_O_4_ spinel coating in 1 wt.% NaCl solution is 6.79 µA/cm^2^, which is significantly lower than the 24.8 µA/cm^2^ of Q235 steel in the same solution. This indicates that the prepared MnFe_2_O_4_ spinel coating exhibits significantly better corrosion resistance than uncoated Q235 steel. By comparing the data in Table 1 and Table 2, we found that the corrosion currents of uncoated Q235 steel in NaCl solutions with concentrations of wt.% = 1%, 2%, 3%, 3.5%, 4%, and 5% are 24.8 µA/cm^2^, 29.4 µA/cm^2^, 53.2 µA/cm^2^, 72.3 µA/cm^2^, 62.5 µA/cm^2^, and 55.7 µA/cm^2^, respectively. In contrast, the corrosion currents of Q235 steel with MnFe_2_O_4_ spinel coating are 6.79 µA/cm^2^, 10.6 µA/cm^2^, 12.4 µA/cm^2^, 15.6 µA/cm^2^, 41.0 µA/cm^2^, and 40.0 µA/cm^2^, respectively, showing reductions of 72.6%, 63.9%, 76.6%, 78.4%, 34.4%, and 28.2%. This indicates that corrosion resistance is improved at each concentration of the corrosive solution. As the concentration of Cl^−^ in the corrosive solution increases, the protective effect of the spinel coating on Q235 steel is somewhat limited. This limitation is likely due to the Cl^−^ ions causing pitting corrosion on the coating, which then progresses to more extensive corrosion. However, compared to uncoated Q235 steel, the MnFe_2_O_4_ spinel coating still provides significant protection to the substrate. The concentration gradient of NaCl corrosive solutions chosen for this study is wt.% = 1, 2, 3, 3.5, 4, and 5, as the Cl^−^ concentration in seawater is usually less than wt.% = 5 NaCl, and in practical industrial production, the Cl^−^ concentration is typically less than wt.% = 3.5 NaCl. Therefore, we believe that the MnFe_2_O_4_ spinel coating can meet the corrosion resistance requirements for steel in coastal industrial and craft applications.

#### 3.3.2. Electrochemical Impedance Spectroscopy Analysis

**Figure 12:**
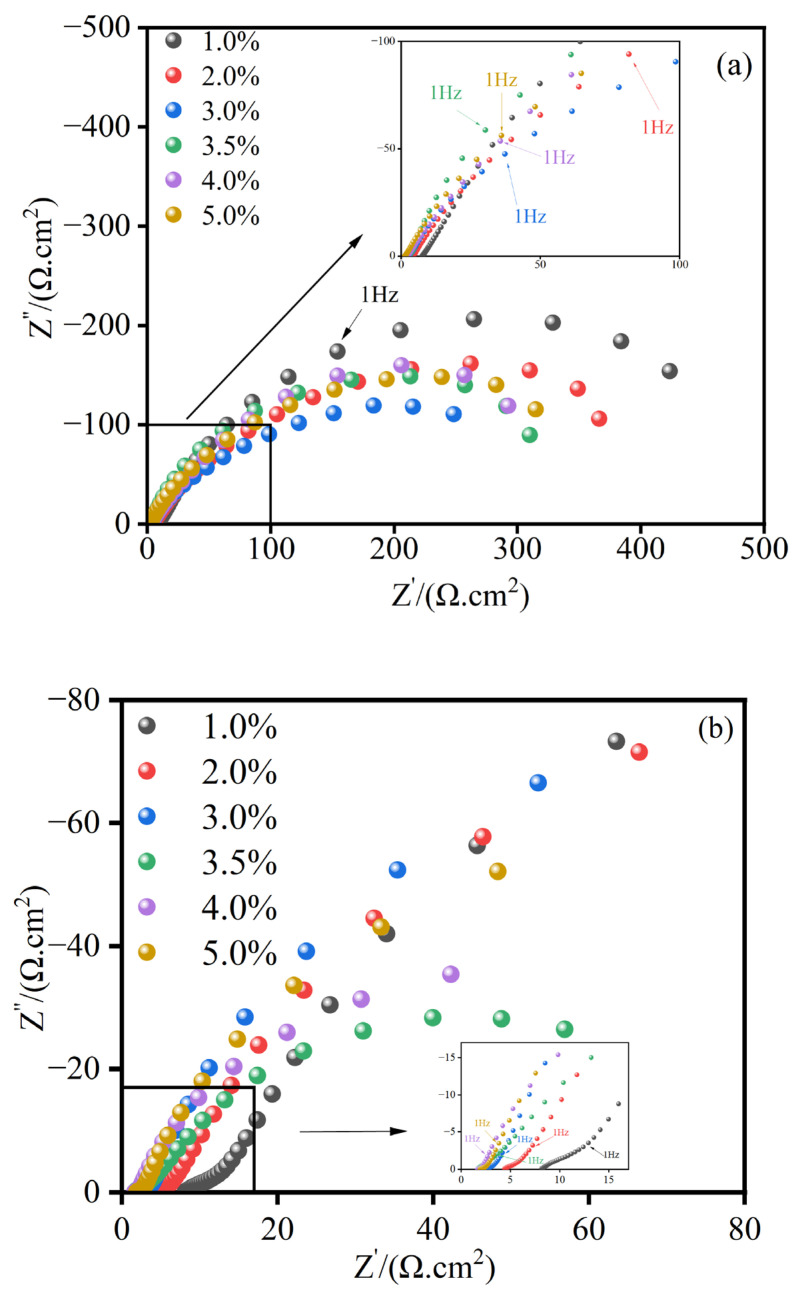
Nyquist curve of samples after 120 min of electrolysis with or without coating in NaCl solution with different mass fractions: 1%, 2%, 3%, 3.5%, 4%, and 5%. (**a**) MnFe_2_O_4_ spinel coatings, (**b**) Q235 steel.

**Figure 13:**
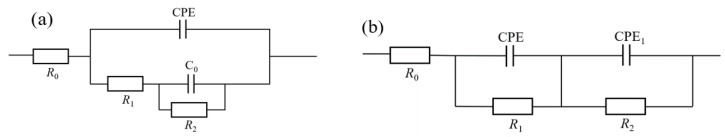
Equivalent circuit diagram of fitting impedance spectrum of different substances. (**a**) MnFe_2_O_4_ spinel coatings, (**b**) Q235 steel.

Figure 12a shows the Nyquist plots for the MnFe_2_O_4_ spinel coating and Q235 steel in NaCl solutions with mass fractions of 1 wt.%, 2 wt.%, 3 wt.%, 3.5 wt.%, 4 wt.%, and 5 wt.%. The fitting of these plots results in the equivalent circuit diagram [39,40,41] shown in Figure 13a and the electrochemical impedance spectroscopy (EIS) fitting data for the MnFe_2_O_4_ spinel coating listed in Table 3. In this circuit, *R*_0_ represents the solution resistance, *R*_1_ represents the coating resistance, *R*_2_ represents the charge transfer resistance, CPE represents the coating capacitance, and *C*_0_ is the double-layer capacitance. Similarly, Figure 12b shows the Nyquist plots for Q235 steel in NaCl solutions with the same mass fractions, with the corresponding equivalent circuit diagram shown in Figure 13b and the EIS fitting data for Q235 steel listed in Table 4. In this circuit, *R*_0_ represents the solution resistance, indicating the resistance between the saturated calomel electrode and Q235 steel, *R*_1_ represents the rust layer resistance, *R*_2_ represents the charge transfer resistance, and CPE represents the capacitance. The impedance response of the Constant Phase Element (CPE) is defined as:

(2) $$Z_{CPE}={\left(j\omega \right)}^{-n}/Y_{0}$$

where *j* is an imaginary unit, *ω* is the angular frequency of the sinusoidal signal, *Y*_0_ is the admittance at *ω* = 1 rad/s (1/|*Z*|), and *n* (0 ≤ *n* ≤ 1) is the exponent. At *n* = 1, CPE behaves as a capacitive component, and at *n* = 0.5, it exhibits Warburg element characteristics, with a pure resistor at 0 [42]. Due to the non-ideal capacitive characteristics of non-uniform electrodes, *C*_0_ substitutes the ideal double-layer capacitance (*C*_0_). The capacitance of CPE, using *ω*_max_ (the frequency at which the imaginary part of the impedance reaches its maximum value), is calculated as follows [43]:

(3) $$C_{CPE}=Y_{0}{\left(\omega_{max}\right)}^{n-1}$$

(4) $$C_{0}=Y_{0}^{1/n}{\left(\frac{1}{R_{1}}+\frac{1}{R_{2}}\right)}^{(n-1)/n}$$

The film is considered a planar capacitor. From Figure 12a, it can be seen that at low NaCl mass fractions, the Nyquist plot initially appears as a straight line, transitioning to a semicircular shape at low frequencies. The MnFe_2_O_4_ spinel coating exhibits similar capacitive arcs in NaCl solutions of varying mass fractions, indicating that changes in the NaCl concentration do not affect the corrosion behavior of the MnFe_2_O_4_ spinel coating. The MnFe_2_O_4_ spinel coating shows a capacitive arc at low frequencies, with the arc radius initially decreasing and then increasing with the NaCl mass fraction, reflecting an inverse trend in corrosion resistance. The radius of the capacitive arc is largest when wt.(NaCl)% = 1. Compared to Figure 12b, the Q235 steel also exhibits capacitive arcs in low NaCl mass fraction solutions. From Table 3, it is evident that the charge transfer resistance *R*_2_ initially decreases and then increases, with the minimum *R*_2_ observed at 3.5 wt.% NaCl. However, compared to Table 4, *R*_2_ for the MnFe_2_O_4_ spinel coating is several hundred times higher, indicating that the MnFe_2_O_4_ spinel coating exhibits varying corrosion resistance in NaCl solutions of different concentrations. This is because the increasing Cl^−^ ions in the corrosive solution challenge the pitting resistance of the MnFe_2_O_4_ spinel coating. Under low Cl^−^ conditions, the corrosion reaction of the spinel coating is less efficient, whereas at higher concentrations, the reaction rate increases. When pitting occurs on the coating surface, more Cl^−^ ions begin to accumulate at the pitting sites, leading to local concentration polarization and accelerating the corrosion reaction from within the pits. However, the MnFe_2_O_4_ spinel coating enhances the corrosion resistance of the substrate to varying degrees at all concentrations.

**Figure 14:**
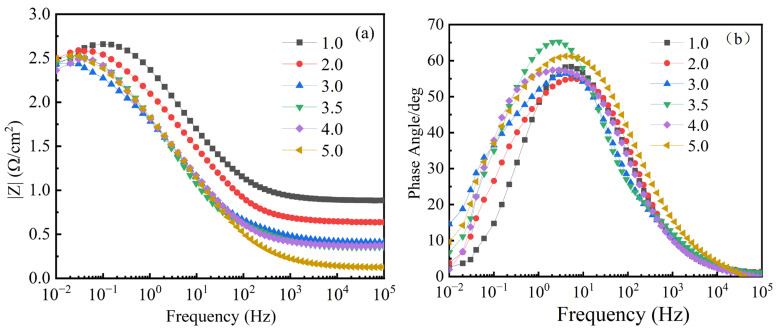
Bode diagram of MnFe_2_O_4_ spinel coating under different mass fractions of NaCl: 1%, 2%, 3%, 3.5%, 4%, and 5%. (**a**) Variation of impedance with frequency, (**b**) Variation of phase angle with frequency.

Figure 14 shows the Bode plots of coated Q235 steel in NaCl solutions with mass fractions of 1 wt.%, 2 wt.%, 3 wt.%, 3.5 wt.%, 4 wt.%, and 5 wt.%. In these plots, Figure 14a represents the variation of impedance with frequency, and Figure 14b represents the variation of the phase angle with frequency. From Figure 14a,b, it can be observed that a capacitive arc appears in the low-frequency region. As the NaCl mass fraction increases, the peak value of the phase angle generally shifts towards the high-frequency region, which explains the improvement in the barrier properties of the coating, suggesting that the coating is more effective in blocking corrosive agents. Furthermore, |Z| decreases with the increase in NaCl concentration. Under the condition of wt. NaCl% = 1, the diameter of the capacitive loop and the absolute value of the impedance |Z| are the highest, indicating that the MnFe_2_O_4_ spinel coating exhibits the strongest corrosion resistance under this condition. In Figure 14a, all curves show little change in |Z| in the high-frequency region, and from Table 3, it can be seen that the change in R_2_ is smaller than the change in R_1_ with varying concentrations of the corrosive solution. This indicates that the corrosion reaction on the surface of the MnFe_2_O_4_ spinel coating is mainly hindered by the accumulation of corrosion products rather than being inhibited by the reaction itself. In Table 4, both R_1_ and R_2_ show significant changes, indicating that uncoated Q235 steel undergoes both diffusion and charge transfer during corrosion. The MnFe_2_O_4_ spinel coating can inhibit the charge transfer process.

**Figure 15:**
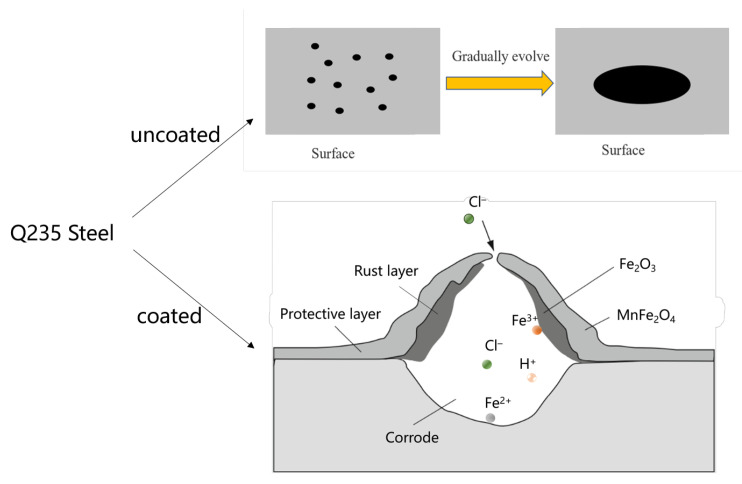
The schematic diagram of the corrosion mechanism.

The corrosion resistance of MnFe_2_O_4_ spinel coatings in NaCl solutions with different mass fractions was investigated. The results of the polarization curves and electrochemical impedance tests indicate that MnFe_2_O_4_ spinel coatings exhibit higher corrosion resistance in low mass fraction NaCl solutions. Notably, in a 1 wt.% NaCl solution, the corrosion current density of the MnFe_2_O_4_ spinel coating is 6.79 µA/cm^2^, which is significantly lower than the corrosion current density of Q235 steel under the same conditions at 24.8 µA/cm^2^. This demonstrates that the MnFe_2_O_4_ spinel coating enhances the corrosion resistance of Q235 steel in NaCl solutions of varying concentrations. A schematic illustration of the corrosion process is presented in Figure 15.

## 4. Conclusions

This thesis primarily addresses the corrosion susceptibility of Q235 steel in atmospheric environments. It proposes a new approach involving the enhancement of rust formation to create a stable rust layer, followed by the in situ preparation of a precursor using the sol-gel method and high-temperature in situ processing to fabricate MnFe_2_O_4_ spinel corrosion-resistant coatings. The preparation process of MnFe_2_O_4_ spinel corrosion-resistant coatings is investigated, and their corrosion resistance is explored using polarization curves, electrochemical impedance spectroscopy, and corrosion weight loss methods. This method has low dependency on equipment, utilizes inexpensive and readily available materials, and can complete the reinforcement treatment of the rust layer on the substrate surface at ambient pressure and relatively low temperatures. Consequently, it significantly enhances the corrosion resistance of corroded Q235 steel. This makes the method applicable to practical industrial production, providing a strategy for the recycling of metals discarded due to corrosion. It can effectively reduce resource waste and environmental pollution. The main conclusions are as follows:

(1) Q235 steel undergoes electrolytic rust enhancement to form a stable rust layer. The precursor can then be prepared in situ using the sol-gel method, and MnFe_2_O_4_ spinel corrosion-resistant coatings can be fabricated through high-temperature in situ processing.
(2) Based on the XRD, FT-IR, morphological patterns of the samples, and cross-sectional morphology characterizations, the optimal conditions for precursor preparation are determined to be a Mn^2+^ concentration of 0.1 mol/L and an electrolysis time of 2 h.
(3) The results of the polarization curves indicate that MnFe_2_O_4_ spinel corrosion-resistant coatings exhibit higher corrosion resistance in low mass fraction NaCl solutions. Notably, in a 1 wt.% NaCl solution, the corrosion current density of the MnFe_2_O_4_ spinel coating is 6.79 µA/cm^2^, which is significantly lower than the corrosion current density of Q235 steel under the same conditions at 24.8 µA/cm^2^. This demonstrates that the MnFe_2_O_4_ spinel coating enhances the corrosion resistance of Q235 steel.
(4) The electrochemical impedance spectroscopy (EIS) results indicate that the MnFe_2_O_4_ spinel corrosion-resistant coating enhances corrosion resistance by inhibiting the charge transfer process. Compared to uncoated Q235 steel, the MnFe_2_O_4_ spinel coating significantly protects Q235 steel in corrosion solutions with wt.% NaCl = 1, 2, 3, 3.5, 4, and 5, exhibiting the strongest corrosion resistance at wt.% NaCl = 1.

## Figures and Tables

**Table 1:** Electrochemical parameters of polarization curves of MnFe_2_O_4_ spinel coating under different mass fractions of NaCl: 1%, 2%, 3%, 3.5%, 4%, and 5%.

NaCl wt.%	MnFe_2_O_4_ Spinel Coating		
	*E*_corr_ (mV)	*I*_corr_ (µA/cm^2^)	*R*_p_ (kΩ·cm^2^)
1.0	−169	6.79	2.330
2.0	−231	10.6	1.860
3.0	−249	12.4	1.830
3.5	−253	15.6.	1.390
4.0	−247	41.0	0.561
5.0	−235	40.0	0.473

**Table 2:** Electrochemical parameters of polarization curves of Q235 steel under different mass fractions of NaCl: 1%, 2%, 3%, 3.5%, 4%, and 5%.

NaCl wt.%	Q235		
	*E*_corr_ (mV)	*I*_corr_ (µA/cm^2^)	*R*_p_ (kΩ·cm^2^)
1.0	−385	24.8	0.857
2.0	−396	29.4	0.586
3.0	−395	53.2	0.518
3.5	−379	72.3	0.292
4.0	−400	62.5	0.351
5.0	−397	55.7	0.497

**Table 3:** MnFe_2_O_4_ spinel coating fitting data of electrochemical impedance spectroscopy under different mass fractions of NaCl: 1%, 2%, 3%, 3.5%, 4%, and 5%.

NaCl wt.%		MnFe_2_O_4_ Spinel Coating				
	*R*_0_/Ω·cm^2^	*R*_1_/Ω·cm^2^	*R*_2_/Ω·cm^2^	CPE/mF·cm^−2^	*n*	*C*_0_/μF·cm^−2^
1.0	7.76	77.7	536	762	0.780	67.4
2.0	4.26	3.08	523	679	0.712	52.4
3.0	2.51	6.37	476	638	0.637	285
3.5	2.25	5.75	466	722	0.721	670
4.0	2.35	17.7	553	709	0.754	44.2
5.0	1.35	8.61	501	712	0.712	127

**Table 4:** Q235 fitting data of electrochemical impedance spectroscopy under different mass fractions of NaCl: 1%, 2%, 3%, 3.5%, 4%, and 5%.

NaCl wt.%		Q235					
	*R*_0_/Ω·cm^2^	*R*_1_/Ω·cm^2^	*R*_2_/Ω·cm^2^	CPE/mF·cm^−2^	*n*_1_	CPE/mF·cm^−2^	*n*_2_
1.0	8.10	301	6.17	804	0.5	500	1
2.0	4.36	260	2.19	778	0.5	500	1
3.0	2.48	216	1.33	831	0.5	500	1
3.5	1.88	90.6	2.41	738	0.5	521	0.982
4.0	1.67	93.6	0.49	819	0.5	500	0.934
5.0	1.61	156	0.86	821	1	500	0.5

## Data Availability

The raw data supporting the conclusions of this article will be made available by the authors on request.
